# A Youth-Centered Digital Infographic on Vaping Risks (What’s in a Vape?): Mixed Methods Study

**DOI:** 10.2196/75694

**Published:** 2025-09-11

**Authors:** Kendra Nelson Ferguson, Kyla Christianson, Adrian Delgado, Gina Martin, Stephanie E Coen, Laura Struik

**Affiliations:** 1 Western University London, ON Canada; 2 University of British Columbia Okanagan Kelowna, BC Canada; 3 Athabasca University Alberta Canada; 4 University of Nottingham Nottingham United Kingdom

**Keywords:** youth vaping, youth-centered digital infographic, health communication, mixed methods

## Abstract

**Background:**

As youth engagement with traditional public health warnings declines, innovative strategies are needed. Visually compelling, youth-driven digital content such as interactive infographics may help bridge knowledge gaps, enhance risk perception, and support more informed decision-making. Despite this potential, limited research has assessed its effectiveness in conveying vaping-related harms to youth.

**Objective:**

To address this gap, this study evaluated the impact of a codeveloped, youth-informed digital infographic (*What’s in a Vape?*) on enhancing vaping education and improving youth understanding of vaping-related harms.

**Methods:**

A convergent parallel mixed methods design was used to assess the impact of a youth-informed digital infographic. The infographic was created in collaboration with youth coresearchers and youth advisory councils to ensure relevance. Participants were recruited through community partners, school boards, and youth networks. By May 2024, we had enrolled 63 high school students aged 14 to 19 years (mean age 16.5, SD 1.2 years) primarily from Ontario and British Columbia. The survey evaluated baseline knowledge of vaping, engagement with the infographic, and postexposure perceptions on whether the content contributed to increased awareness or understanding of vaping.

**Results:**

Data collection took place between April 2024 and May 2024. Quantitative analysis showed that 87% (55/63) of participants agreed that the infographic effectively communicated key information, and 86% (54/63) gained new knowledge about vaping. In addition, 73% (46/63) found that the infographic was presented in an easy and meaningful way, whereas 52% (33/63) indicated that they would definitely share it with others, reflecting strong engagement. However, over half (33/63, 52%) also found the amount of information excessive, and 17% (11/63) found it difficult to digest, indicating variation in youth information preferences. Thematic analysis of qualitative feedback revealed four key themes: (1) the visual content enabled gaining new insights into and knowledge of vaping, (2) the visual design had a positive impact on engagement with information, (3) sourced information enhanced the credibility of the infographic information, and (4) the digital design of the infographic made complex information more understandable. Qualitative insights contextualized and supported the quantitative findings, highlighting both benefits and areas for improvement.

**Conclusions:**

This study demonstrates that youth-driven digital infographics may serve as useful health communication tools. Findings highlight the importance of peer-led design; evidence-based content; and interactive, visually compelling formats in enhancing youth comprehension and receptiveness to health messaging. By integrating youth feedback into development and prioritizing digital engagement, the infographic bridged knowledge gaps while reinforcing the credibility of its content. Variability in feedback about content overload suggests that future versions should consider more layered or modular designs. Results suggest that such approaches may complement broader public health strategies to curb youth vaping and inform future educational interventions. Continued research is warranted to assess long-term impacts on attitudes and behavior.

## Introduction

### Background

Youth vaping remains a critical public health concern in Canada, with surveillance data showing a consistent rise since 2017 [1]. Although overall prevalence rates have remained relatively stable in recent years, the frequency of use has risen, signaling a shift toward habitual use and potential nicotine dependence among youth [2]. Vaping among youth has been linked to a range of well-documented health risks, including neurological, respiratory, and cardiovascular effects [2-6]. It is also associated with increased likelihood of substance use (eg, cannabis, cocaine, and heroin) [7-10] and higher rates of mood disorders such as anxiety and depression [7]. Despite these harms, many youths remain unaware of the risks, particularly those related to device design, heavy metal exposure, and addiction potential [11-14].

This gap in awareness emphasizes the need for clearer and more effective communication strategies. Rohde et al [15] found that youths recognized risks such as respiratory issues and device explosions but were less aware of the harms related to skin contact with e-liquids, exposure to diacetyl, cardiovascular damage, carcinogenic compounds, and addiction. Furthermore, Greiner Safi et al [16] found that uncertain language in youth-targeted public health messaging can lead to confusion or the dismissal of the information altogether, reinforcing the importance of clear and accessible messaging.

To improve message clarity and engagement, public health campaigns must prioritize effective, youth-aligned communication strategies. Visual aids such as infographics have been shown to simplify complex health information, reduce literacy barriers, and support knowledge retention [17], particularly when paired with direct messaging that can influence health behaviors [17]. Nonetheless, public health messaging has been slow to adopt these approaches, with many campaigns overlooking interactive digital tools despite their relevance to youth [18,19]. Studies by Rohde et al [15], England et al [20], and Stalgaitis et al [19] highlight the value of digital platforms in expanding the reach and relevance of health messaging.

Beyond format, actively involving youth in campaign development is increasingly recognized as essential for creating messages that resonate. Incorporating youth perspectives enhances the relevance, trust, and effectiveness of health communication [16]. For example, the Rethink Vape campaign [20] exemplifies the benefits of this approach, demonstrating that youth-centered campaigns lead to greater message retention and impact. Participants who engaged with the campaign demonstrated greater comprehension of vaping-related information, heightened risk perception, and stronger intentions to avoid vaping. As youth engagement with traditional public health warnings declines, incorporating youth-driven, visually compelling content with interactive digital strategies may be critical in addressing high vaping rates [16].

Given these findings, digital infographics codeveloped with youth offer a promising but underexplored avenue for bridging knowledge gaps, enhancing perceived relevance, and promoting informed decision-making. While large-scale campaigns such as The Real Cost [18] have demonstrated effectiveness, these efforts were primarily adult led and evaluated using broad exposure metrics rather than participatory design approaches. Even campaigns with participatory designs, such as Rethink Vape, focused on short-term engagement and lacked formal evaluation of individual infographic components [20].

### Objectives

In contrast, this study assesses the effectiveness of a youth-informed, codeveloped digital infographic (ie, *What’s in a Vape?*) created through an iterative design process with youth coresearchers. By delivering the infographic via an interactive web interface and assessing it using a convergent mixed methods approach, this study uniquely captured both perceived impact and experiential feedback. By integrating quantitative and qualitative findings, we offer a nuanced, youth-centered perspective on the role of digital infographics in vaping education.

## Methods

### Infographic Development

This project began with a poll conducted among 3 youth advisory councils, each composed of high school–aged youth (n=2, 67% in British Columbia and n=1, 33% in Ontario) to gather their questions about e-cigarettes and how they wanted them answered. *What’s in a vape?* emerged as the most pressing unanswered question, and all councils advocated for an interactive, digital infographic to address it. The development of the infographic was led by a youth coresearcher (AD), an undergraduate student in the School of Nursing at the University of British Columbia Okanagan, who began by conducting a literature review on current evidence related to vape components and their health effects. Following this, AD engaged in collaborative sessions with the senior author (LS) to organize the findings into a mind map, visually structuring key information before transitioning to an infographic. Mind mapping served as an initial step to systematically categorize vaping-related risks, ensuring a logical and comprehensive infographic design. To transition this information to a digital, web-based infographic, AD collaborated with a design thinking researcher, who used Figma (Figma, Inc), a collaborative interface design tool, to incorporate graphic design and interactive digital strategies (eg, clickable links and illustrations), ultimately creating the final product. The infographic was hosted as a web-based interactive resource accessible via a unique hyperlink. This web-based format was chosen for its ease of access, scalability, and compatibility across devices commonly used by young people (eg, smartphones, tablets, and laptops). Once the initial draft was completed, feedback was gathered from 2 high school youth coresearchers. Their input led to multiple refinements. A follow-up poll of the youth advisory councils was conducted to collect additional thoughts, opinions, and suggestions for improvement. After final revisions, the infographic was ready for fielding (Multimedia Appendix 1).

### Study Design

This study used a mixed methods approach. Specifically, a convergent parallel design was used whereby both quantitative and qualitative data were collected simultaneously, analyzed as separate but complementary datasets, and converged for interpretation in the Discussion section [21]. Following independent analyses, the research team compared findings across both data types to identify areas of convergence and divergence. For example, descriptive statistics (eg, perceived knowledge gain and clarity of presentation) were examined alongside qualitative themes (eg, what knowledge was gained and what aspects supported clarity) to deepen interpretation. This integrative process allowed for a more nuanced understanding of how youth experienced and responded to the infographic.

A cross-sectional web-based survey, the Youth Vaping Information Project (VIP), was developed and distributed to assess perceptions of the interactive digital infographic—*What’s in a Vape?*—which was embedded within the survey. This study was reported according to the Checklist for Reporting Results of Internet E-Surveys [22].

### Ethical Considerations

Ethics approval for this study was obtained in February 2024 from the Human Research Ethics Boards at both the University of British Columbia (H23-03957) and Western University (124387). Upon completion of the survey, participants were emailed a CAD $10 (US $7.26) gift card as a thank you for their time.

### Recruitment and Participants

The research team disseminated the Youth VIP recruitment poster to community partners (eg, health units and school boards), youth advisory councils, and youth coresearchers via email, requesting their support in circulating the poster to their networks. The poster included a QR code and web link directing potential participants to the Youth VIP survey on Qualtrics (Qualtrics International Inc). The survey began with a letter of information outlining study details and emphasizing that participation was voluntary and that participants could opt out at any time. This was followed by an eligibility questionnaire and assent or consent questions. Eligibility criteria were being a resident of Canada, currently enrolled in high school (grades 9-12), and fluent in English. Youth aged <16 years were required to obtain parental consent in addition to their own assent.

Recruitment followed a purposive, self-selection approach, primarily reaching youth with existing connections to school- or community-based programming. This strategy was intentionally chosen to engage youth who are more likely to interact with health messaging and digital platforms, aligning with the infographic’s participatory development process and web-based delivery format. Given the focus on youth-driven content, it was important to include youth who could provide informed, critical feedback on the design and content of the infographic. However, we acknowledge that this approach may have introduced sampling bias as it likely excluded less engaged youth or those not involved in structured programming. As such, the sample may not be representative of all youth populations.

While the sample included participants from Ontario and British Columbia, data on school type (eg, public or private), rural or urban setting, or other contextual characteristics were not collected. Following demographic and behavioral questions, participants were shown the *What’s in a Vape?* infographic, which was embedded in the Qualtrics survey platform. They were encouraged to take their time in reviewing the infographic to ensure that they could accurately respond to the subsequent questions. The survey included a hyperlink that directed participants to view the interactive version of the infographic on a separate web page before returning to complete the survey questions. This version included clickable sections and embedded visuals created using Figma.

The final sample consisted of 63 participants (n=26, 41% identified as boys, n=35, 56% identified as girls, and n=2, 3% preferred not to disclose) aged 14 to 19 years (mean age 16.5, SD 1.2 years). The sample was geographically concentrated in Ontario (n=34, 54%) and British Columbia (n=29, 46%), and 44% (28/63) of the participants identified as White.

### Data Collection

The survey was developed by a multidisciplinary team (KNF, AD, and LS) specializing in health promotion, health behavior, health geography, and nursing. Questions were informed by knowledge translation principles [23] to ensure an accurate evaluation of the infographic. After drafting the survey, 2 high school youth coresearchers reviewed and refined the questions for youth relevance and project alignment. Following minor adjustments, members of 3 youth advisory councils piloted the survey for readability, functionality, and usability. The survey was finalized after further discussions and feedback from the councils. Data were collected between April 2024 and May 2024. Participants who provided consent or assent and parental consent (if aged <16 years) completed the survey, which took 20 to 30 minutes.

### Survey

#### Measures

The survey questions captured participant demographics (ie, age, sex, gender, ethnicity, and socioeconomic status), and participants responded to a series of closed-ended questions assessing vaping behaviors and exposure to vape education. Questions related to vape use included the following—“Have you ever used a vape?” “Do you currently vape?” “Have you attempted to quit vaping?”—with *yes* or *no* response options. The latter 2 questions—“Do you currently vape?” and “Have you attempted to quit vaping?”—were only presented to participants who answered *yes* to "Have you ever used a vape?" Exposure to vape education was assessed using the following questions—“Have you attended any vape education presentations at school?” and “Have you noticed any educational material related to vaping at school (eg, posters in the hall)?”—also using *yes* or *no* response options. After viewing the infographic, participants provided feedback in four areas: (1) intentions to share the infographic (“Will you share knowledge from this infographic with your family/friends?”; response range: *definitely not* to *definitely yes*), (2) effectiveness in addressing knowledge gaps (“The infographic tells me what I need to know about vapes”; response range: *strongly disagree* to *strongly agree*), (3) clarity and presentation (“The infographic is presented in an easily digestible and meaningful way”; response range: *strongly disagree* to *strongly agree*), and (4) information sufficiency (response options: *too much*, *the right amount*, or *not enough*).

To gain deeper insights into participants’ perceptions and experiences with the infographic, the survey included 4 open-ended questions designed to elicit qualitative feedback. These questions were included after participants viewed the infographic and explored whether the content contributed to increased awareness or understanding of vaping as well as the overall appeal of the infographic, while also prompting participants to reflect on its design and content. Specifically, participants were asked the following: (1) “Does the infographic provide new knowledge? If yes, what did you learn?” (2) “What I liked about the infographic was...” (3) “What I did not like about the infographic was...” (4) “What would you change about the infographic?”

#### Quantitative Analysis

Descriptive statistics (frequencies and proportions) were calculated using Apple Numbers (version 3.6.2; Apple Inc), a spreadsheet application for Macintosh computers, which was used solely for basic data summarizations given the exploratory nature of the analysis. No inferential statistical tests were conducted.

#### Qualitative Analysis

Thematic analysis was conducted to explore the open-ended survey responses. Using an inductive approach, 2 researchers (AD and LS) independently reviewed and grouped responses into themes based on recurring ideas [24]. Any irrelevant responses (eg, *N/A*, *I don’t know*, or *none*) were removed. Following this, AD and LS acted as “critical friends” [25] to challenge and refine theme interpretations. Key discussions led to revisions of theme names to ensure alignment with participant perspectives. For example, the theme *visual content enabled gaining new insights and knowledge into vaping* was initially titled *gaining new knowledge on vaping*, but after discussions, it was revised to further reflect the nuances of youth experiences. Once consensus was reached, the first author (KNF) reviewed the themes for plausibility and consistency, strengthening the study’s rigor and trustworthiness.

## Results

### Overview

A narrative contiguous approach was implemented for reporting and integrating the mixed methods results. In this approach, quantitative findings are presented first, followed by qualitative findings, with each set of results outlined in different subsections [26]. Both datasets are then synthesized to inform the overall discussion.

### Quantitative Results

Table 1 provides the demographic and behavioral characteristics of the sample (N=63). Of the participants, 46% (28/61) identified as male, and 54% (33/61) identified as female, and by gender, 43% (26/61) identified as boys, and 57% (35/61) identified as girls. Ethnic representation was predominantly White (28/61, 46%), followed by East Asian (15/61, 25%). Socioeconomic status varied, with 21% (13/63) never worrying about money, 44% (28/63) worrying only about extras, and 35% (22/63) having just enough or not enough to get by. Most participants rated their physical and mental health as very good (23/63, 37% and 24/63, 38%, respectively) or good (21/63, 33% and 19/63, 30%, respectively). Among participants who reported using a vape, 41% (26/63) indicated that they currently vaped. Of those current vapers, 65% (17/26) reported having attempted to quit. Social exposure to vaping was high, with 35% (22/63) indicating that many, most, or all of their friends vaped; 44% (28/63) reporting that some vaped; and 21% (13/63) reporting that none of their friends vaped. The most common exposure settings included school (44/63, 70%), peers or friends (29/63, 46%), and online or social media (26/63, 41%), with less frequent exposure at home and the workplace (4/63, 6% each). Just over half of the participants had attended vape education presentations at school (34/63, 54%) or had noticed vape education materials at school (33/63, 52%).

**Table 1:** Characteristics of the sample (N=63).

Variable		Values
Age (y), mean (SD; range)		16.5 (1.2; 14-19)
**Sex, n/N (%)**		
	Male	28/61 (46)
	Female	33/61 (54)
	Missing	2/63 (3)
**Gender, n/N (%)**		
	Boy	26/61 (43)
	Girl	35/61 (57)
	Missing	2/63 (3)
**Race or ethnicity, n/N (%)**		
	Black	3/61 (5)
	East Asian	15/61 (25)
	Indigenous	7/61 (11)
	South Asian	2/61 (3)
	Southeast Asian	4/61 (7)
	West Asian or Middle Eastern	2/61 (3)
	White	28/61 (46)
	Missing	2/63 (3)
**Socioeconomic status, n/N (%)**		
	Never worry about money	13/63 (21)
	Only worry about money for extras	28/63 (44)
	Just enough or not enough money to get by	22/63 (35)
**Physical health, n/N (%)**		
	Excellent	12/63 (19)
	Very good	23/63 (37)
	Good	21/63 (33)
	Fair or poor	7/63 (11)
**Mental health, n/N (%)**		
	Excellent	8/63 (13)
	Very good	24/63 (38)
	Good	19/63 (30)
	Fair or poor	12/63 (19)
**Vaping experience, n/N (%)**		
	Currently vape	26/63 (41)
	Tried to quit vaping	17/26 (65)
**Friends who vape, n/N (%)**		
	Many, most, or all	22/63 (35)
	Some	28/63 (44)
	None	13/63 (21)
**Setting in which they were most exposed to vaping (participants could select all that applied), n/N (%)**		
	At school	44/63 (70)
	Online (social media)	26/63 (41)
	With peers or friends	29/63 (46)
	At home	4/63 (6)
	At the workplace	4/63 (6)
	Activities	11/63 (17)
**Attended vape education presentations at school, n/N (%)**		
	Yes	34/63 (54)
	No	29/63 (46)
**Noticed vape education materials at school, n/N (%)**		
	Yes	33/63 (52)
	No	30/63 (48)

Table 2 presents closed-ended responses to the infographic. Most participants (55/63, 87%) agreed or strongly agreed that the infographic effectively conveyed the necessary information. Regarding presentation, 73% (46/63) of the participants agreed or strongly agreed that the infographic was presented in an easy and meaningful way, whereas 17% (11/63) expressed disagreement or strong disagreement. When asked about the amount of information provided, 52% (33/63) felt that there was too much information, whereas 46% (29/63) believed that the amount was appropriate. A substantial proportion of respondents (54/63, 86%) reported gaining new knowledge from the infographic. In terms of sharing the infographic with family and friends, 52% (33/63) indicated that they would definitely do so.

**Table 2:** Closed-ended infographic-related responses (N=63).

Infographic-related questions		Participants, n (%)
“**It tells me what I need to know”**		
	Strongly agree or agree	55 (87)
	Neutral	4 (6)
	Disagree or strongly disagree	4 (6)
“**It is presented in an easy and meaningful way”**		
	Strongly agree or agree	46 (73)
	Neutral	6 (10)
	Disagree or strongly disagree	11 (17)
**Amount of information**		
	Too much	33 (52)
	The right amount	29 (46)
	Not enough	1 (2)
**Gained new knowledge**		
	Yes	54 (86)
	No	9 (14)
**Likely to share it with family and friends**		
	Definitely	33 (52)
	Maybe	23 (37)
	Probably or definitely not	7 (11)

### Qualitative Results

#### Overview

Thematic analysis of open-ended survey responses revealed four main themes: (1) the visual content enabled gaining new insights into and knowledge of vaping, (2) the visual design had a positive impact on engagement with information, (3) sourced information enhanced the credibility of the infographic information, and (4) the digital design of the infographic made complex information more understandable. While open-ended survey responses were initially disaggregated by vaping status (ever vapers or never vapers), they were ultimately merged due to substantial thematic overlap between the groups. Themes and illustrative quotes were consistent across both user types, suggesting similar patterns of infographic engagement and perceived knowledge gain. Each theme is explored through participant quotes from both ever and never vapers.

#### The Visual Content Enabled Gaining New Insights Into and Knowledge of Vaping

As reflected in the quantitative closed-ended questions, most of the participants reported gaining new knowledge from the infographic. Many participants noted that they had previously focused on the substances inside vapes rather than the materials used to construct them. One participant explained the following:

I did not know about the materials of a vape; we mostly learn about the substances inside rather than the carrier.

Never vaper

Others found the infographic useful in clarifying how e-cigarettes function, with one participant stating that it “provides an explanation for how e-cigarettes work” (ever vaper). Beyond understanding the mechanics of vaping devices, participants also reported learning about the overall health risks associated with vaping. Several noted that they were unaware of how vaping affects the respiratory system and academic performance, and how metals from vapes accumulate in vital organs such as the brain, liver, and kidneys. This is highlighted in the following quote—“[I learned that] inhaling chemicals and nicotine can damage lung function, leading to breathing difficulties” (ever vaper)—whereas another participant mentioned learning about how “aluminum particles are collected in the brain, liver, and kidneys” (never vaper). Along these lines, participants also reported increased awareness of the chemicals and metals in vape liquids and aerosols. One ever vaper put it as follows:

I learned that e-cigarette liquids contain nicotine, flavouring agents, and chemicals harmful to our health.

Another participant echoed this sentiment:

I learned about the different harmful metals and substances found in vapes, which I didn’t know of before.

Never vaper

It is apparent that the infographic provided valuable insights into both the components of vapes and the associated health risks for both ever and never vapers alike.

#### Positive Impact of Visual Design on Engagement With Information

Participants expressed an appreciation for the visual elements of the infographic, highlighting how the design effectively communicated essential information. The vibrant visuals helped capture attention, with one participant mentioning that “the infographic’s vibrant colors and engaging design immediately caught [my] attention, making the information more accessible and enjoyable to explore” (never vaper). Similarly, another participant added that “the visual aspect made it appealing and more inviting to read” (ever vaper).

In addition to its appeal, participants found that the infographic’s structure made the content easier to personalize and more accessible. One participant explained that it “allows users to customize the display of infographics to their personal preferences” (never vaper), whereas another participant said that “it was scientific, and had a diagram which helped me understand; it was also well organized” (ever vaper). The responses demonstrate that the visual aspects of the infographic were perceived to enhance both the accessibility of and engagement with the information.

#### Sourced Information Enhanced the Credibility of the Infographic Information

It was evident that youth participants valued the credibility and transparency of the information presented, particularly regarding the associated risks of vaping and the components of vapes. Pointing to the effective communication of health risks, one participant shared that “all the info about the harms is impactful and helps you to realize the effects of vaping on your body” (ever vaper).

Similarly, a never vaper commented on learning about “all the ingredients in e-cigarettes, all the damage they cause to the human body, the names of the ingredients and what they do.” Another participant added the following:

I liked how the infographic contained information about the parts of a vape and also the harmful effects of vaping.

Ever vaper

Participants also discussed the importance of the infographic’s transparency when it comes to the sources used and provided reference materials. This is explained in the following quote:

I like the source and reference materials of this information diagram the most so that I can learn more about electronic cigarettes.

Ever vaper

This was complemented by another participant, who highlighted the inclusion of data and statistics:

The inclusion of statistics and data adds credibility to the information presented in the infographic.

Never vaper

#### The Digital Design of the Infographic Made Complex Information More Understandable

Most participants recognized that the infographic effectively broke down complex information into digestible and understandable content. One participant shared the following:

[It was] easy to understand even though there was lots of new scientific information on how a vape works.

Ever vaper

Another participant echoed this sentiment by saying the following:

[It made] complex information easier to understand through data visualization.

Never vaper

The clear labeling and detailed presentation were helpful, as highlighted in the following quote:

I liked how it pointed to specific parts of the vape and broke it down. I liked how it was clearly labelled as well.

Never vaper

In addition, the infographic’s visuals, such as diagrams and illustrations, were particularly appreciated for their ability to simplify the information presented. One participant shared the following—“the amount of diagrams provided to illustrate the main point of the texts [was helpful]” (ever vaper)—and another added the following—“I like that it uses charts and diagrams to display data, making it easier to understand and remember” (never vaper). These visual elements, combined with detailed information, made the content both accessible and engaging, helping participants understand the different aspects of vaping in a clear and concise manner.

## Discussion

### Principal Findings

This study explored whether a codeveloped, youth-informed digital infographic can enhance vaping education and strengthen understanding of vaping-related harms among youth. By integrating quantitative and qualitative data, this study provided comprehensive insight into how youth interact with and interpret vaping-related educational materials. To our knowledge, this is one of the first studies to examine the impact of a youth-created digital infographic on youth vaping, offering insights into how peer-designed digital materials can influence young people’s knowledge and engagement with vaping-related education. Although the findings were presented using a narrative contiguous approach, deeper integration of the quantitative and qualitative data reveals a strong convergence between youth-reported outcomes and the content of their open-ended responses. Quantitative results established the prevalence of knowledge gain and infographic engagement, and qualitative findings offered illustrative context and nuance to these patterns.

The infographic’s youth-centered and youth-created design may have enhanced its relevance and appeal, supporting previous research findings that peer-led interventions can be effective tools for disseminating vaping-related information [16,27]. The iterative process with youth allowed for the refinement of infographic concepts to better align with the needs and preferences of a youth audience, reflected in our qualitative findings related to visual content and digital design. As noted by England et al [20], concepts favored by adults are often dismissed by teenagers, emphasizing the need to prioritize youth input in health communication. Given that youth are experts in their own lives, they are well positioned to identify the information most relevant to them [20]. Our findings support the importance of incorporating youth voices and perspectives when developing public health education materials, thereby increasing their potential effectiveness at translating health information.

Findings indicate that participants appreciated the infographic’s ability to simplify complex information, a sentiment echoed by 86% (54/63) of the participants, who reported gaining new knowledge after viewing the infographic. Qualitative responses substantiated this self-reported knowledge gain, with participants noting that the infographic effectively communicated technical facts by breaking down intricate details about vaping risks into digestible and understandable content without overwhelming the audience. Unlike traditional approaches that often oversimplify health information, resulting in vague and unhelpful messaging that may fail to engage or inform youth effectively [16,27], the infographic provided nuanced, evidence-based details in a structured and visually engaging format. This approach acknowledges that young audiences are capable of processing detailed health information when presented in a manner that does not sacrifice depth and accuracy. Rather than reducing content to simplistic messages, the digital format enabled a layered presentation of information, allowing youth to connect with key points. Research has shown that digital platforms, particularly those using interactive and visual elements, enhance youth engagement and comprehension by allowing them to explore content at their own pace [17,19]. Our findings reinforce existing research stating that youth are more responsive to health messaging delivered through digital media as such platforms align with their media consumption habits [18,19].

However, it is important to consider the interpretive limitations of these results. While participants’ responses suggest strong engagement and perceived knowledge gain, these findings are based on self-reported impressions rather than validated knowledge or behavioral outcomes. This limits the extent to which we can infer actual understanding or educational impact. As such, the results should be interpreted as exploratory, offering insight into youth engagement rather than conclusive evidence of learning or behavior change.

The ability of the infographic to translate complicated health information also aligns with research demonstrating that visual and interactive educational tools improve knowledge, particularly for complex topics [17]. Participants stated that they valued the clarity and organization of the information, which is consistent with existing evidence that visually compelling materials enhance comprehension and accessibility [17,28,29]. This aligns with the 73% (46/63) of participants who agreed or strongly agreed that the infographic was presented in an easy and meaningful way, reinforcing that the visual design supported understanding across the sample. Specific design elements such as clear diagrams, attractive colors, and structured layouts were praised for making the content easier to digest, echoing studies that highlight the role of visual aids in improving the retention and clarity of health messages [17,29]. In addition, immersive features such as clickable links and illustrations further enhanced interest, demonstrating the potential of digital platforms to appeal to youth.

While most participants found the infographic engaging and informative, it is important to acknowledge that 52% (33/63) reported the amount of information to be excessive and 17% (11/63) did not find the content easily digestible. These findings suggest that, despite the overall positive sentiment regarding the infographic, some youths may have experienced cognitive overload or difficulty processing the information. This highlights the importance of striking a balance between depth and simplicity when designing digital health tools for young people. Future iterations may benefit from modular formats or interactive layering that allows users to control how much information they engage with at one time, aligning better with diverse learning preferences and capacities.

Beyond design-related considerations, participants also valued the infographic’s credibility and transparency, particularly the inclusion of data and references related to the health effects of vaping. This emphasizes the need for evidence-based, transparent messaging in educational resources as young audiences are often skeptical of health information that lacks clear scientific backing [16,27]. Due to the absence of clear public health messaging on the harms of e-cigarette use, youth are often exposed to mixed messages about vaping risks [20,30-32]. A reduced perception of risk, combined with a general underestimation of nicotine’s addictive properties, can increase youth’s susceptibility to vape marketing strategies [30], contributing to vaping initiation [15]. Thus, the inclusion of scientific sources strengthened the infographic’s credibility among youth and may encourage them to seek out evidence-based materials when evaluating other forms of vaping information.

### Strengths and Limitations

This study has several strengths. First, it contributes to the limited research on youth-centered vaping education by evaluating a digital infographic codeveloped with youth. By incorporating youth perspectives into its design, this study ensured that the educational material was relevant and engaging for the target audience (ie, youth). Second, using a mixed methods approach allowed for a more comprehensive understanding of how youth interact with and interpret vaping-related information. Including both ever vapers and never vapers provided valuable insight into differences in knowledge, perceptions, and responses to educational materials. Finally, the combination of both quantitative and qualitative responses provided a nuanced understanding of how youth interpret and perceive vaping-related messaging.

Despite these strengths, several limitations constrain the interpretive weight of our findings. The sample was geographically limited to Ontario and British Columbia and, due to the small sample size, is not representative of youth in those regions or reflective of the broader diversity of perspectives across Canada. Although efforts were made to include a range of participants, the demographic distribution does not capture the breadth of lived experiences across socioeconomic, cultural, and regional contexts. Contextual details such as school type (eg, public, private, or alternative), rural versus urban location, and other educational or community characteristics were not systematically collected. These gaps restrict our ability to assess how different environments may have shaped youth engagement with the infographic. As vaping prevalence, attitudes, and access to vaping products can vary across provinces, particularly between rural and urban settings [33,34], the findings should be interpreted as exploratory rather than generalizable.

This study also used a purposive self-selection sampling strategy, which primarily reached youth already engaged in school-based programs or youth advisory councils. This aligns with the participatory nature of the intervention, ensuring feedback from youth familiar with digital content and public health messaging. However, it likely introduced selection bias as these participants may differ from less engaged youth in terms of media literacy, health knowledge, or responsiveness to educational tools. As a result, the findings may overestimate perceived relevance or impact and cannot be extrapolated to disengaged or marginalized youth populations. Broader outreach strategies will be necessary to examine the infographic’s effectiveness across different demographic and psychosocial contexts.

Perhaps most significantly, this study relied exclusively on self-reported perceptions to assess knowledge gain and the effectiveness of the infographic. Without validated pre- and postexposure testing or behavioral measures, we cannot determine whether participants’ perceived understanding translated into actual knowledge uptake or shifts in intent to vape. These subjective impressions, although valuable for design feedback, are vulnerable to biases such as social desirability bias, confirmation bias, and imprecise recall. This substantially limits the strength of any claims regarding impact. Future research should incorporate validated, objective assessments of pre- and postexposure knowledge and behavioral intention measures to more rigorously evaluate the impact of youth-centered digital health interventions, including their potential for sustained knowledge retention and behavior change over time.

Finally, while ever vapers and never vapers were both included in the sample, subgroup comparisons were not conducted due to the small sample size. Qualitative responses were initially disaggregated but were ultimately merged due to thematic consistency. However, this decision may have obscured important differences in how youth with different vaping histories interpret and respond to prevention materials. The absence of stratified analyses in the quantitative results similarly limits insight into demographic or behavioral variability. Future studies with larger and more diverse samples should conduct subgroup comparisons (eg, by vaping status, ethnicity, gender, or geography) and control for key variables to better understand how targeted educational tools function across different youth populations.

### Conclusions

This study suggests that youth-driven infographics may be useful educational tools for conveying vaping-related information and enhancing youth engagement with prevention messages. The findings highlight the potential value of participatory design, visual accessibility, and credibility in improving the relevance and clarity of digital health communication for young people. These findings are promising; however, the results should be interpreted with caution due to the small, nonrepresentative sample and reliance on self-reported perceptions rather than objective or behavioral outcomes. While the infographic appeared to support understanding of vaping-related harms among participants, further research is needed to assess its effectiveness with more diverse youth populations and to determine whether such tools can lead to sustained knowledge retention or behavior change over time.

## Appendix

Multimedia Appendix 1 What’s in a Vape? infographic.

## Abbreviations

VIP: Vaping Information Project
