# Supplementary figures and images for: A Youth-Centered Digital Infographic on Vaping Risks (What’s in a Vape?): Mixed Methods Study
Source: JMIR Form Res. 2025 Sep 11;9:e75694. doi: 10.2196/75694 (PMC12464500; doi:10.2196/75694)

**Appendix: “What’s in a Vape?” Infographic**

**
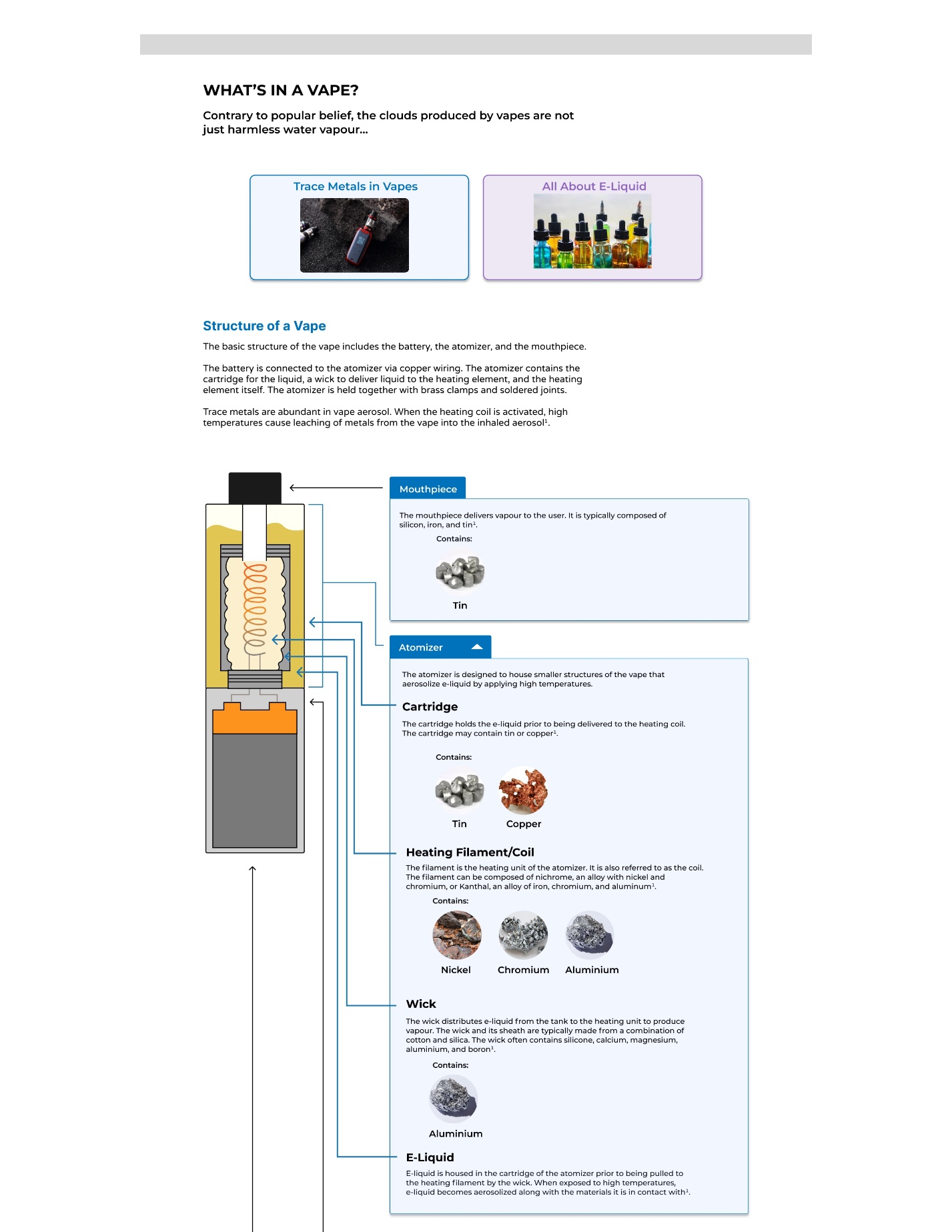
**

**
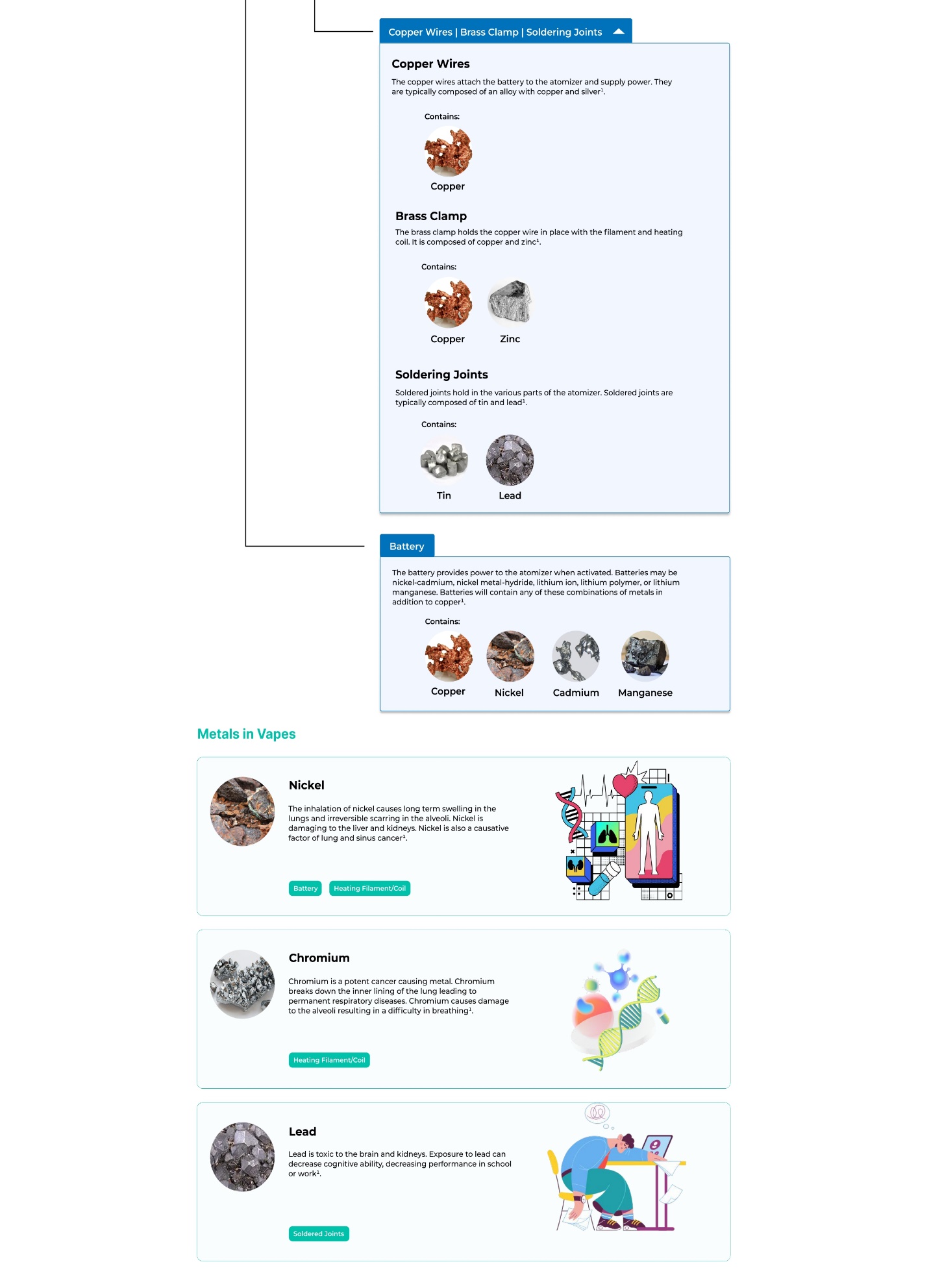
**

**
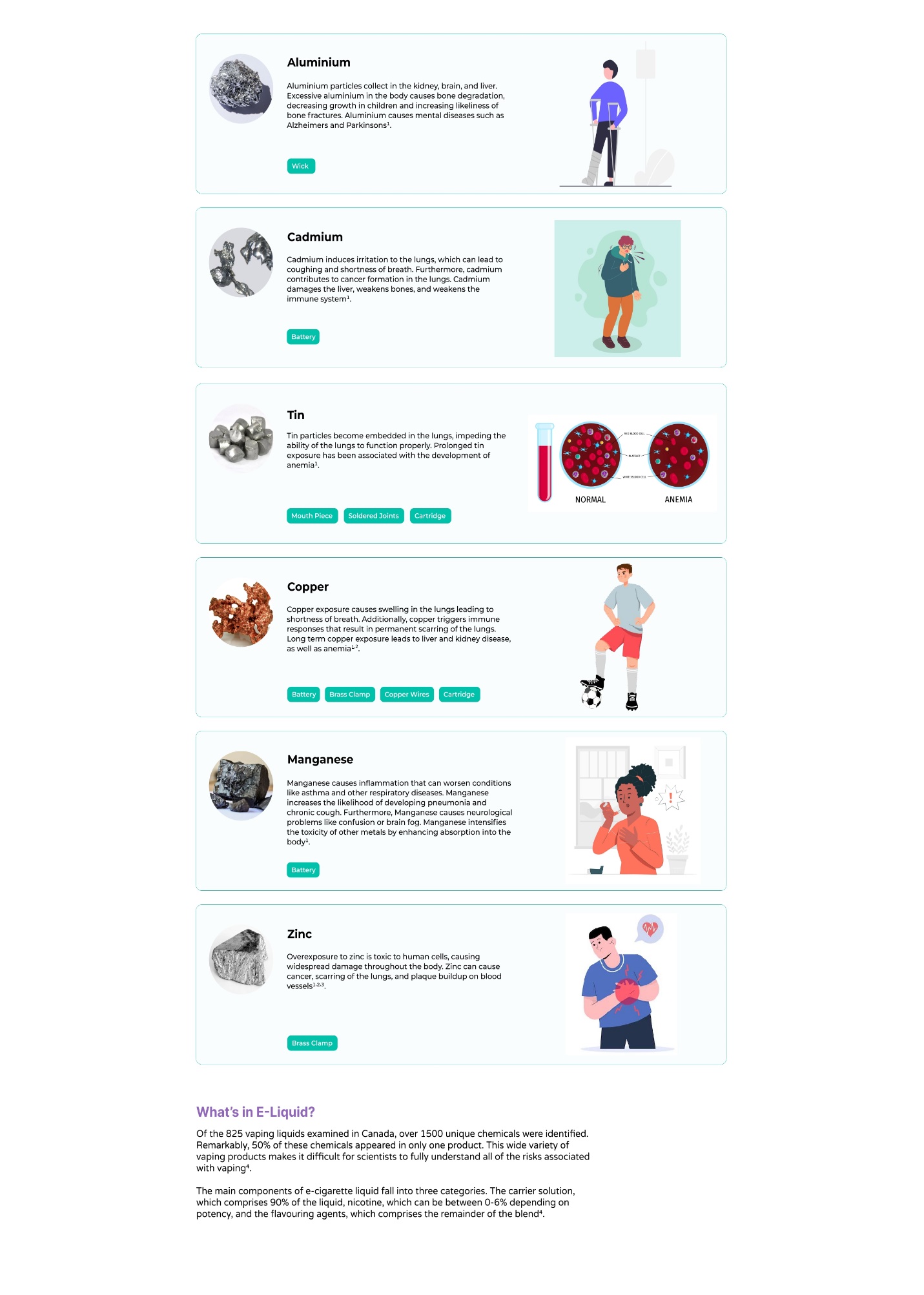
**

**
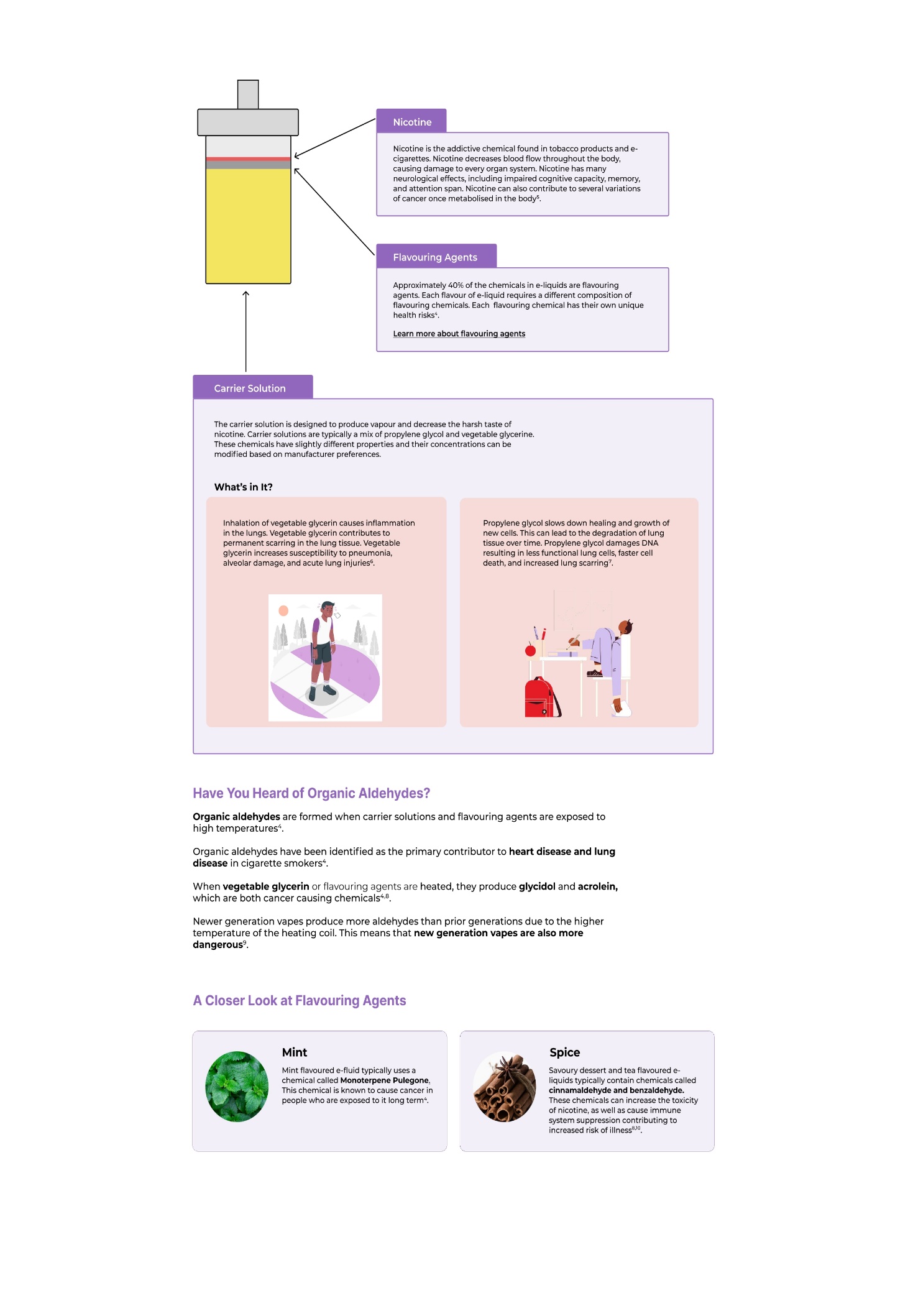
**

**
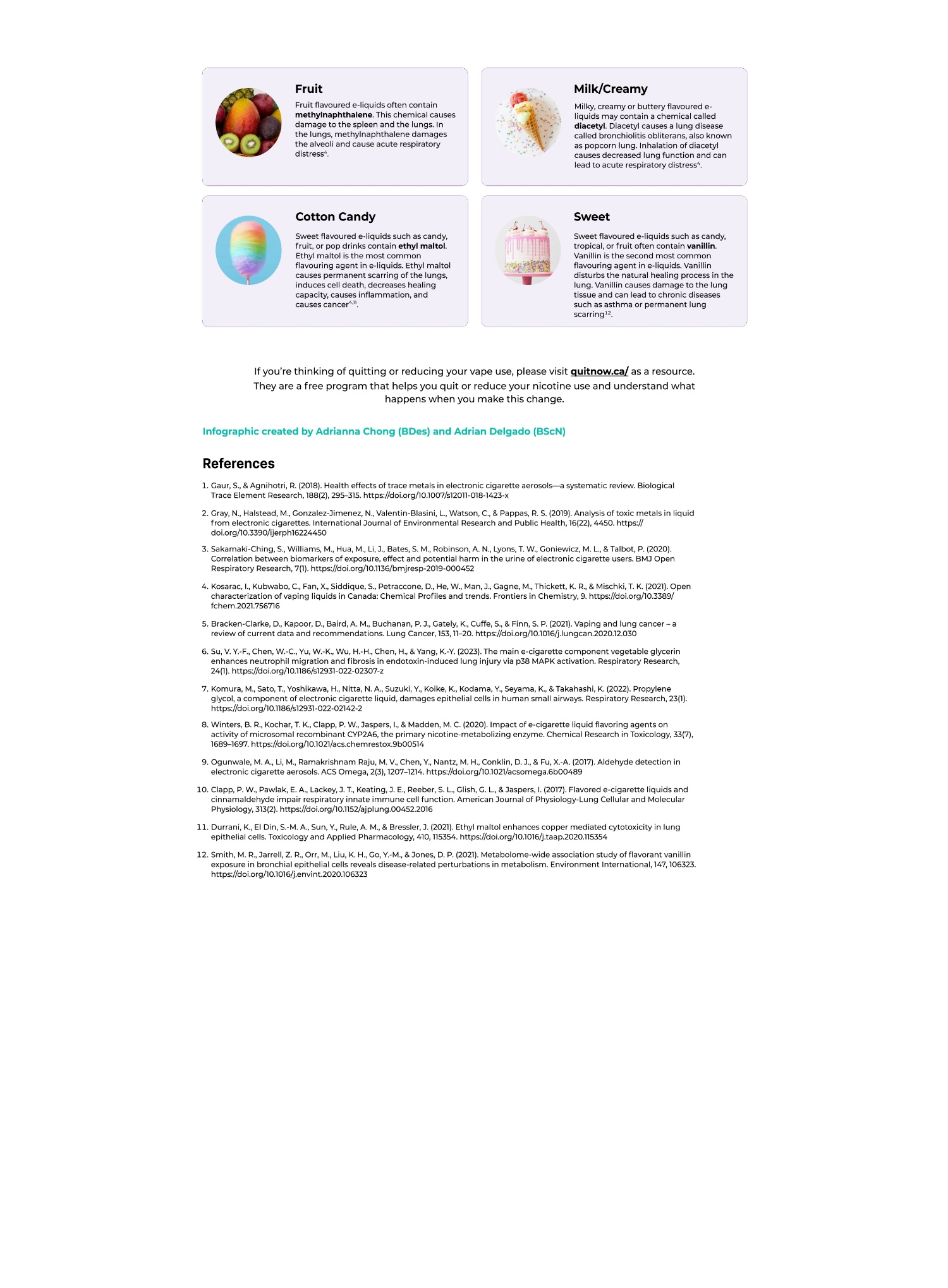
**

Supplement: Multimedia Appendix 1 [file formative_v9i1e75694_app1.docx]
